# Case Report: From concept to care and knowledge translation: developing a community-oriented family health center in a resource-limited post-conflict setting in Sri Lanka

**DOI:** 10.3389/fmed.2026.1895556

**Published:** 2026-10-22

**Authors:** Antony Sheron Vethanayagam, Kumaran Subaschandren, Kaarunnya Parthipan, Sakana Kannathasan, Saman Pathirana

**Affiliations:** 1 Center for Digital Epidemiology, Faculty of Medicine, University of Jaffna, Jaffna, Sri Lanka; 2 Department of Community and Family Medicine, Faculty of Medicine, University of Jaffna, Jaffna, Sri Lanka; 3 Provincial Department of Health Service, Jaffna, Sri Lanka

**Keywords:** community-oriented primary care, family medicine, participatory action research, primary health care, Sri Lanka

## Abstract

Primary Health Care (PHC) oriented systems in resource-limited, post-conflict settings face compounded challenges arising from population ageing, rising non-communicable diseases, workforce constraints, and weakened community trust. In Sri Lanka's Northern Province, decades of conflict followed by economic instability and the COVID-19 pandemic further exposed limitations of fragmented, facility-centered Primary Care. This paper presents a concept-driven community case study describing the progressive development of a community-oriented Family Health Center (FHC) in Northern Sri Lanka, tracing its evolution from a small FHC to a nationally recognized and internationally connected Primary Care, education and research hub. The model integrates Family Medicine, Community-Oriented Primary Care (COPC) and Participatory Action Research (PAR) within a single operational framework. Family Medicine provides the clinical and ethical foundation, COPC introduces defined population responsibility with embedded Patient and Public Involvement and Engagement (PPIE) and Community Engagement and Involvement (CEI), while PAR functions as a system-learning engine enabling continuous adaptation and co-creation of knowledge. Locally recruited Community Health Workers (CHWs) operate these principles by linking households, services and research. The FHC delivers comprehensive Primary Care services and supports multilevel education, continuous professional development and capacity building, while functioning as a platform for global collaboration, including a 10,000-participant community cohort supported through digital health systems and international partnerships such as the NIHR DIGIPATHS program. This experience demonstrates how locally grounded PHC-oriented system components can be integrated into health systems and support the transformation in post-conflict settings can evolve into sustainable centers of excellence and trusted global implementation hubs, offering transferable lessons for strengthening PHC-oriented system in comparable contexts.

## 1 Background: why primary care transformation requires integrated partnerships?

Primary Health Care (PHC)-oriented system is widely recognized as the foundation of equitable and resilient health systems. Yet, in resource-limited and post-conflict settings, PHC-oriented systems frequently remain fragmented, reactive, and insufficiently responsive to the long-term needs of communities. Sri Lanka exemplifies this paradox: despite universal free healthcare and strong public health achievements, disparities persist in Primary Care access, continuity, and quality particularly in historically marginalized regions such as the Northern Province, Sri Lanka (1–3).

Three decades of armed conflict in Northern Sri Lanka disrupted social cohesion, displaced communities, weakened institutional capacity, and eroded trust in public systems. Post-conflict recovery occurred alongside demographic ageing, rising non-communicable diseases (NCDs), workforce shortages, and economic instability. These pressures were further magnified by the COVID-19 pandemic, which disrupted routine care and exacerbated chronic disease morbidity (4, 5).

While national reforms such as the World bank funded Primary Health Systems Strengthening Project (PSSP) emphasize people-centered care, continuity, and digital integration, implementation at the frontline level remains challenging (6). In this context, facility-based or disease-specific interventions are insufficient. What is required is a system-oriented, relational, and learning-driven approach that integrates service delivery, community participation, education, and research.

This manuscript describes such an approach: the progressive development of a community-oriented FHC in Jaffna District, Northern Sri Lanka. Importantly, while the partnership is rooted in national and subnational collaboration, the model evolved to engage global academic research and education partnerships, demonstrating how locally grounded Primary Care platforms can mature into internationally credible knowledge-generating systems.

## 2 Methodology

### 2.1 Study design and approach

This study employs an implementation case study design informed by PAR principles. Rather than evaluating static and single time intervention, the manuscript documents the progressive and multi-phase transformation of a Primary Care Health Center into an integrated service, education and research hub between 2012 and 2026.

### 2.2 Setting and timeframe

The implementation center was situated at the FHC in Kondavil, Jaffna District, Northern Province of Sri Lanka, a region undergoing long term reconstruction following three decades of armed conflict. The observation period covers three distinct developmental phases spanning 2012 to 2026 (Table 1).

**Table 1:** Key development phases and milestones of family health center: 2012–2026.

Phase & timeframe	Primary focus	Key operational milestones	Data sources & evidence
Phase 1: Foundation (2012–2016)	Service reliability & clinical practice	Establishment of Family Medicine clinics at Kondavil Initial clinical profiling of acute and chronic local disease burdens	Clinical encounter logs, baseline community audits
Phase 2: Integration (2017–2021)	COPC & community mobilization	Recruitment and deployment of CHWs Launch of Patient & Public Involvement (PPIE/CEI) forums Emergency COVID-19 response (24-hour helpline, household monitoring)	CHW outreach logs, community feedback logs, epidemic response records
Phase 3: Expansion (2022–2026)	Digital infrastructure & global research	Establishment of 10,000-participant EHR community cohort Biobank creation (6,000 + bio-samples) Launch of international partnerships (e.g., NIHR DIGIPATHS)	EHR databases, biobank registries, peer-reviewed publications and grant awards

### 2.3 Data sources and documentation

Evidence supporting the implementation process, service adaptations and outcome observations was synthesized from multiple administrative, clinical and research data streams:

- Administrative and clinical records: Monthly clinic attendances, electronic health record (EHR) registry entries and CHWs household visit logs.
- Participatory reflection logs: Documented reflection and action cycles from regular joint meetings involving clinicians, CHWs, academic leads and community representatives.
- *Programmatic evaluations and publications:* Findings from over 20 peer—reviewed research paper, doctoral dissertations and subnational policy evaluation reports (Supplementary Table S1) generated through the platform.

## 3 Timeline table

### 3.1 Conceptual framework: integrating three complementary models

The FHC was intentionally built on three integrated conceptual foundations (Figure 1): Family Medicine, Community-Oriented Primary Care (COPC) and Participatory Action Research (PAR) (7). These frameworks were not applied sequentially but woven together to form a single operational system.

**Figure 1:**
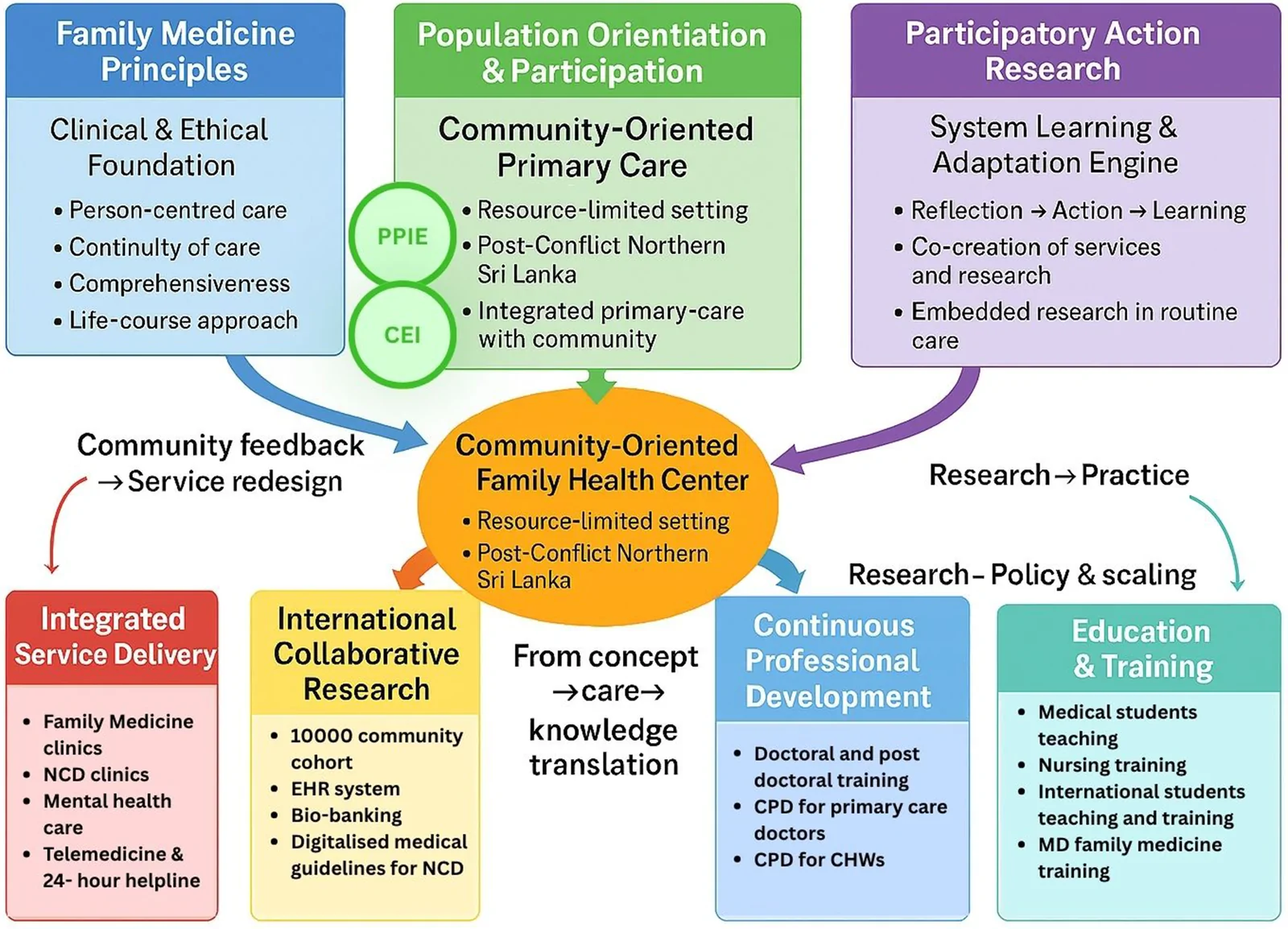
Integration of three complementary models within the community-oriented Family Health Center.

#### 3.1.1 Principles of family medicine: clinical continuity and ethical grounding

Family Medicine provided the clinical foundation of the FHC. Its principles continuity, comprehensiveness, person-centeredness, and contextual care are particularly relevant in post-conflict environments, where fragmented care and episodic encounters undermine trust and effectiveness (2, 8, 9).

At Kondavil village, Family Medicine enabled holistic management of acute illness, chronic disease, mental health, geriatric care, and preventive services within enduring Doctor family community relationships. This continuity proved critical for managing multi-morbidity and psychosocial distress rooted in conflict-related adversity (10).

#### 3.1.2 Community-oriented primary care: population responsibility with participation

COPC extended Family Medicine beyond individual clinical encounters to encompass defined population responsibility, enabling a shift from reactive, facility-based care toward proactive, community-responsive Primary Care (1, 11). Through systematic community profiling, participatory needs assessment and iterative service planning, COPC facilitated alignment of Primary Care services with locally relevant priorities in a resource-limited, post-conflict context.

Through CEI and PPIE, communities participated across clinical care, education, and research, contributing to service planning, patient education materials, and the development of community cohort studies. These processes enabled communities to evolve from passive recipients of care into knowledge partners, enhancing trust, relevance and sustainability of Primary Care interventions in a post-conflict setting.

Crucially, COPC at the FHC explicitly embedded Patient and Public Involvement and Engagement (PPIE) and Community Engagement and Involvement (CEI) as foundational mechanisms rather than consultative or time-limited activities. These processes were operationalized through structured community engagement forums and a locally recruited Community Health Worker (CHW) network, ensuring that service design, delivery and evaluation were co-produced with communities rather than externally imposed (3, 8).

CHWs formed the operational bridge between households, the health center, and the wider health system. Recruited through participatory community processes, CHWs functioned as community representatives with clearly defined roles aligned with WHO guidance, while remaining adapted to post-conflict realities. Their work included subfertility care (12, 13), connecting community with Primary Health Care services (14, 15), home based follow-up for older adults, medication adherence support for chronic disease patients, maternal and child health outreach, cancer screening awareness and early identification of psychosocial distress. During the COVID-19 pandemic, CHWs played an expanded role in risk communication, monitoring of vulnerable households and linkage to a 24-hour helpline, demonstrating system resilience.

#### 3.1.3 Participatory action research: embedding learning in practice

PAR served as the system learning engine. PAR enabled cyclic reflection, action and adaptation, transforming routine service delivery into a source of continuous improvement and ethical knowledge generation (5, 16).

Through PAR, patients, CHWs, students, clinicians and researchers functioned as co-creators of knowledge, dissolving traditional boundaries between care and research. This approach was particularly suited to environments where randomized or rigid research designs are neither feasible nor culturally appropriate. Significantly, beyond service delivery, CHWs actively contributed to PAR cycles by supporting data collection, facilitating community feedback and participating in reflection sessions. This dual role as service extenders and knowledge producers strengthened both care delivery and system learning.

## 4 From a small family health center to community-oriented primary care

The FHC began as a modest FHC serving a rural, post-conflict population of approximately 11,500 people. Early assessments revealed limited community awareness of services, low engagement in chronic disease self-management, overcrowding and fragmented record-keeping (3).

By progressively integrating COPC and PAR principles, the FHC transitioned into a community-oriented FHC, characterized by:

- Defined catchment responsibility,
- CHW-led outreach and follow-up,
- Community participation in service planning,
- Alignment of services with population needs.

During the COVID-19 pandemic, this integrated structure enabled rapid adaptation, including the establishment of a 24-hour helpline and continuity of care for high-risk patients, demonstrating resilience and community trust (4).

## 5 Four interconnected outcome domains of the model

As illustrated in Figure 1, the integrated model generated four mutually reinforcing outcome domains.

### 5.1 Integrated service delivery

The FHC delivers comprehensive Primary Care services including:

- Family Medicine clinics
- NCD and metabolic clinics
- Mental health and post-conflict psychosocial care
- Geriatric and home-based care
- Telemedicine and digital follow-up

These services are continuously refined through community feedback and PAR cycles, ensuring responsiveness and relevance (11).

### 5.2 Education and multilevel training

Education became a core function of the FHC:

- Undergraduate medical education for medical students
- Clinical teaching for students from SingHealth—DukeNUS universities
- Allied health and nursing education and training
- MD Family Medicine training, with the FHC recognized as a Center of Excellence
- PhD and post-doctoral research training

This integration ensured that learning occurred within real-world Primary Care contexts rather than isolated academic settings (1, 16, 17).

### 5.3 Continuous professional development and capacity building

Continuous professional development was embedded within service delivery, focusing on:

- CPD for doctors and nurses
- Family Medical team training
- Cardio-metabolic, renal and chronic disease care
- Leadership and digital skills

Capacity building extended beyond individuals to include research governance, data management, and digital epidemiology, strengthening institutional sustainability (18).

### 5.4 Research, knowledge translation and global collaboration

Demonstrated service reliability, community trust and ethical governance enabled the FHC to engage in global research partnerships, most notably through collaboration with the National Institute for Health and Care Research (NIHR).

Key milestones include:

- A 10,000-participant community cohort using electronic health records
- A biobank of over 6,000 bio-samples
- Mixed-methods and implementation studies on atrial fibrillation and cardiovascular risk
- Launch of the DIGIPATHS program, supporting digitally integrated care pathways for multiple long-term conditions in Primary Care (19)

These partnerships illustrate how locally grounded Primary Care platforms can mature into internationally trusted research environments, even in resource-limited post-conflict settings.

While the FHC originated through national and subnational collaboration, its sustained integration of service delivery, community engagement, education and governance enabled the gradual development of meaningful global academic and research partnerships. These collaborations emerged organically as international institutions recognized the FHC as a credible, ethically governed, community-embedded Primary Care platform.

Key partnerships include collaboration with the National Institute for Health and Care Research (NIHR, UK), culminating in the DIGIPATHS program for digitally integrated care pathways; academic collaboration with SingHealth through undergraduate medical education, faculty exchange and capacity building; and engagements with partners focusing on implementation science, workforce development and Primary Care strengthening.

Additional collaborations span digital health, water and environmental health research, cancer prevention, reproductive and subfertility care and cardiovascular disease prevention, reflecting the breadth of community-identified priorities addressed through the FHC. These partnerships underscore how a locally grounded Primary Care model can evolve into an international implementation and learning hub, without reliance on high-cost infrastructure.

## 6 Impact and transferable lessons

The evolution of the FHC from a rural clinic into a globally connected research hub demonstrates how PHC-oriented system components can be integrated into health systems and support the transformation in the resource-limited and post-conflict settings. Rather than a rigid template, this model offers a flexible framework adaptable to other low-resource contexts.

### 6.1 Community and systemic impact

Integrating Family Medicine, COPC and PAR yielded immediate clinical, structural and academic gains. At the community level, the model improved healthcare access, empowered patient trust and ensured continuity of care in a post-conflict rural population. Systemically, the center empowered the local health workforce by recruiting and training CHWs who acted as vital links between households and clinical care. This CHWs improved health literacy supported chronic disease self-management and facilitated not only the traditional health care provision but also the early identification of psychosocial distress. In the aspect of academic and research globalization, the FHC established itself as a national center of excellence for Family Medicine training, larger international grants and epidemiological cohorts generating over 20 peer-reviewed publications and directly informing subnational health policies.

### 6.2 Practical lessons and phased implementation

Initial efforts focused on delivering dependable Family Medicine services to treat acute and chronic illnesses, thereby establishing baseline clinical credibility within the community on achieving clinical stabilization, establishing reliable patient tracking for major NCDs and earning community trust.

Once clinical operations stabilized, the center expanded into population-based profiling, PPIE channels and structured CHW outreach to acquire specific systemic readiness thresholds: a fully operational CHW network covering target households, mature community engagement structures capable of co-designing initiatives and established PAR cycles embedded in routine health care delivery.

This community-oriented PHC enabled the operational and ethical benchmarks to recognize our FHC as the national training center for the Family Medicine discipline. Further, the recognition of national center of excellence and the triangulation of CEI, PPIE and PAR based service delivery gratified global academic and research entities on larger cohort studies, establishment of bio bank system, digital epidemiological interventional studies and international medical clinical placements.

## 7 Discussion

This community case study illustrates how the structural transformation of a local health facility into a community-oriented FHC can reorient healthcare delivery in a post-conflict and resource constrained setting. By transitioning from a reactive model focused on acute care to a continuous, proactive system centered on Primary Care, the facility addresses the rising burden of NCDs and chronic conditions in Northern Sri Lanka.

### 7.1 Health system transformation and community engagement

Rebuilding healthcare delivery in post-conflict regions requires addressing disrupted infrastructure, fractured institutional trust and a longstanding shortage of specialized health personnel (20). Our experience shows that embedding Primary Care within the local community creates an accessible entry point that reduces overcrowding at tertiary hospitals while improving continuous management of chronic conditions like atrial fibrillation and hypertension (21, 22). Essential to this transition is PPIE. Actively engaging patients and local stakeholders strengthens health literacy, reduces social stigma surrounding chronic and mental health conditions and fosters institutional trust as a vital requirement for sustainable healthcare delivery in post-war communities (3).

### 7.2 International perspectives and comparative insights

The structural evolution observed in Northern Sri Lanka aligns with PHC-oriented system transformation models across other post-conflict and resource limited settings globally:

- *Community-embedded care networks:* Comparable to Iran's khaneh behdasht model, staffed by behvarz community health workers, embedding multidisciplinary Primary Health teams directly within rural communities drastically improves early NCD detection, preventive screening and continuous follow-up care among underserved populations (23).
- *Family Health strategy frameworks:* Brazil's *Estratégia Saúde da Família* (ESF) demonstrated that decentralizing Primary Care through team-based Family Health units significantly reduces preventable hospitalizations and bridges health equity gaps (24). Our findings reinforce that localized adaptations of Family Medicine frameworks yield similar operational efficiencies in South Asian health systems.
- *Post-conflict reconstruction:* Experiences from Primary Care reconstruction in Rwanda (25) and Cambodia (26) demonstrate that health system strengthening serves a dual purpose: expanding essential health service delivery while simultaneously restoring social cohesion and public trust in civic institutions.

### 7.3 Strengths and limitations

While this community case study illustrates a promising model for transforming Primary Care within a post-conflict context, several inherent limitations must be acknowledged.

#### 7.3.1 Methodological limitations

First, as a descriptive implementation case study, observations concerning community trust, patient empowerment and service acceptability rely predominantly on qualitative feedback, administrative records and reflective logs beyond controlled experimental trials. Furthermore, the fluid and adaptive nature of PAR meant it was not feasible to establish a parallel control population or a nonintervention baseline facility for direct statistical comparison. Long term health economic metrics and longitudinal clinical outcome tracking were beyond the scope of this study and warrant further investigation.

#### 7.3.2 Generalizability limitations

Generalizability to other settings may also be constrained. The FHC benefited considerably from its close institutional proximity to the Faculty of Medicine at the University of Jaffna, which provided essential academic leadership, clinical support and human resources. Replicating this framework in remote Primary Care settings lacking embedded university support, academic leadership or institutional resources may present distinct challenges.

## 8 Future directions

Future efforts should focus on multi center implementation trials to evaluate the scalability of this model across other provinces in Sri Lanka. Integrating digital health registers, formalizing bidirectional referral pathways and conducting rigorous cost effectiveness analyses will be crucial to inform national health policy and support sustainable scaling across low-resource Primary Care systems.

## 9 Conclusion

This manuscript presents a comprehensive community case study of how a small FHC in a resource-limited, post-conflict setting evolved into a community-oriented FHC capable of delivering integrated care, strengthening the health workforce, educating future professionals and generating globally relevant knowledge. The transformation was not the result of a single intervention, but of intentional integration of principles of Family Medicine, COPC and PAR, with Patient and Public Involvement and community engagement embedded throughout.

The experience demonstrates that Primary Care transformation is fundamentally relational. Trust-based partnerships with communities, sustained engagement with academic institutions and adaptive leadership enabled the FHC to navigate uncertainty, respond to crises such as COVID-19 and progressively expand its reach from local service delivery to international collaboration and policy relevant research (27).

While deeply shaped by the Sri Lankan post-conflict context, the model offers transferable principles for other countries: phased development, embedded learning systems, co-production with communities and integration of service, education and research at the Primary Care level. These principles are particularly relevant for health systems seeking to strengthen Primary Care in fragile, underserved or transitioning contexts.

In an era of growing global interest in people centred and digitally enabled Primary Care, this community case study illustrates that resource-limited settings can lead innovation, not merely adapt it. The community-oriented FHC provides a practical blueprint for how local care platforms can become engines of resilience, equity and global knowledge translation.

## Data Availability

The original contributions presented in the study are included in the article/Supplementary Material, further inquiries can be directed to the corresponding author.
