# Supplementary material for: Case Report: From concept to care and knowledge translation: developing a community-oriented family health center in a resource-limited post-conflict setting in Sri Lanka
Source: Front Med (Lausanne). 2026 Oct 22;13:1895556. doi: 10.3389/fmed.2026.1895556 (PMC13737576; doi:10.3389/fmed.2026.1895556)
Supplement: Supplementary file 1 [file Table1.docx]

**Supplementary Table S1 - Summary of local and international collaborative research publications in Northern Sri Lanka via COPC integrated FHC**

| **No.** | **Theme / Domain** | **Citation** | **Study focus & relevance** |
| --- | --- | --- | --- |
| I | Cardiovascular Health & Pathways | Vethanayagam AS, Gooden TE, Uruthirakumar P, et al. The pathway to diagnosis and follow-up care for atrial fibrillation in Sri Lanka: a descriptive longitudinal study. *NIHR Open Res*. 2023. | Maps clinical pathways and patient journeys for atrial fibrillation (AF) diagnosis and management in Sri Lanka. |
| II | Digital Health & Diagnostics | Vethanayagam AS, Surenthirakumaran R, Gooden TE, et al. Diagnostic accuracy of digital technologies compared with 12-lead ECG in the diagnosis of atrial fibrillation in adults: a systematic review protocol. *PLOS ONE*. 2024. | Protocol evaluating point-of-care digital screening tools vs standard 12-lead ECG. |
| III | Health System Perspectives | Sheron VA, Shanmugathas S, Gooden TE, et al. Healthcare provider and patient perspectives on access to and management of atrial fibrillation in Northern Sri Lanka. *BMC Health Serv Res*. 2022. | Qualitative assessment of system barriers and enablers in post-conflict primary care delivery. |
| IV | Global Health Generalisability | Gooden TE, Wang J, Goulart AC, ..., Sheron VA, et al. Generalisability of and lessons learned from a mixed-methods study conducted in three low- and middle-income countries to identify care pathways for atrial fibrillation. *BMJ Glob Health*. 2024. | Cross-country comparative analysis (Brazil, China, Sri Lanka) on AF care integration in LMICs. |
| V | Population Epidemiology Protocol | Kanesamoorthy S, Sheron VA, Uruthirakumar P, et al. Prevalence of atrial fibrillation in Northern Sri Lanka: study protocol. *BMJ Open*. 2022;12:e062489. | Protocol for establishing population-based prevalence metrics in the Northern Province. |
| VI | Health Economics & Screening | Kanesamoorthy S, Sheron VA, Uruthirakumar P, et al. Cost-effectiveness of three screening strategies for atrial fibrillation in Sri Lanka: a decision-tree modelling analysis using community-based prevalence data. *BMJ Glob Health*. 2026;11(3):e019592. | Economic evaluation determining optimal community screening models for resource-limited settings. |
| VII | Systematic Reviews | Kanesamoorthy S, Sheron VA, Uruthirakumar P, et al. The impact of rate and rhythm control strategies on quality of life for patients with atrial fibrillation: a protocol for a systematic review. *Syst Rev*. 2023;12(1):52. | Systematic review evaluating clinical management strategies on patient quality of life. |
| VIII | Reproductive & Women's Health | Sivalingarajah R, Kesavan V, Coonghe PA, Vethanayagam SA, Jeyaluxmy S, Dhushyanthy K, Paramanathan S. The Prevalence of Vitamin D Deficiency and Associated Factors Among Subfertile Women: An Initial Experience From Northern Sri Lanka. *Cureus*. 2024;16(10):e72094. | Clinical profiling of vitamin D deficiency among women in Northern Sri Lanka. |
| IX | Non-Communicable Disease Burden | Kanesamoorthy S, Nageswaran T, Sriskantharasa K, Uruthirakumar P, Sheron VA, Thayaparan C, Thayakaran R, Scholfield S, Surenthirakumaran R, Sreeharan N, Subaschandren K, Nirmalan M. A cross-sectional analytical study comparing the prevalence of cardiovascular risk factors in two culturally linked communities in post-war Sri Lanka. *J Hypertens*. 2025. | Epidemiological comparison of cardiovascular risk factors in post-war populations. |
| X | Global Health Policy | Krittayaphong R, Phrommintikul A, Bahuleyan CG, Surenthirakumaran R, Namboodiri N, Makkar N, Lordson J, Vethanayagam AS, Uruthirakumar P, Subaschandran K, Kaneshamoorthy S, Lip GYH. Global voices on atrial fibrillation: South/Southeast Asia. *Heart Rhythm O2*. 2024;5(10):687–692. | Regional policy perspective on AF burden and primary care management in South/Southeast Asia. |
| XI | Health Services Delivery | Gooden TE, Wang J, Goulart AC, Vethanayagam SA, Wang H, Varella AC, Paschoal E, Uruthirakumar P, Kanesamoorthy S, Shanmugathas S, Zhang H, Zhong J, Feng M, Li X, Guruparan M, Kumarendran B, Nirantharakumar K, Lip GYH, Thomas GN, Bensenor IM, Rajendra S, Greenfield S, Manaseki-Holland S. Inefficiencies identified in healthcare professional-to-patient handover practices for atrial fibrillation: a mixed-methods study in Brazil, China and Sri Lanka. *BMJ Glob Health*. 2025;10(11):e017517. | Multi-country analysis of clinical communication and patient handovers in primary care. |
| XII | Cardiovascular Risk Studies | Santos IS, Lotufo PA, Goulart AC, ..., Sheron VA, et al. Cardiovascular Health and Atrial Fibrillation or Flutter: A Cross-Sectional Study from ELSA-Brasil. *Arq Bras Cardiol*. 2022;119(5):724–731. | Multi-center collaborative cohort study evaluating cardiovascular health metrics. |
| XIII | Cerebrovascular Epidemiology | Kumaran S, Shribavan K, Powsiga U, Sheron VA, Jency Vinuja HM, Arasalingam A. The epidemiology of stroke in Northern Sri Lanka: A population-based descriptive cross-sectional study. *Sri Lanka J Neurol*. 2025;12(1):20–23. | Population-based baseline metrics on stroke incidence and prevalence in Northern Sri Lanka. |
| XIV | Post Conflict Mental health | Kumaran S, Surenthirakumaran R, Coonghe PAD, et al. Deliberate self-harm and associated psychosocial stressors in post-war Northern Sri Lanka: a community-based study. *Jaffna Med J*. | Epidemiological assessment evaluating rates and social determinants of deliberate self-harm and suicide attempts in post-conflict Jaffna communities. |
| XV | Global Health Partnerships | Lip GYH, Guo Y, Lotufo P, Surenthirakumaran R, Humphreys L, Thomas GN, NIHR Global Reach AF Investigators. The National Institute for Health Research (NIHR) Global Health Research Group on Atrial Fibrillation management. *Eur Heart J*. 2019;40(36):3005–3007. | Foundational overview of international research partnership for AF management across UK, Sri Lanka, China, and Brazil. |
| XVI | Primary Care Health Utilisation | Doherty S, Sureshkumar DS, Thayakaran R, Surenthirakumaran R. Characteristics and Influencing Factors of Healthcare Utilization in Post-conflict Primary Care Attendees in Northern Sri Lanka. *Front Health Serv*. 2022;1:719617. | Empirical analysis of healthcare seeking patterns and out-of-pocket costs at primary care level. |
| XVII | Global Health Policy & Regional NCD Burden | Krittayaphong R, Kaolawanich Y, Phrommintikul A, Guruparan M, Subaschandren K, et al. The burden of atrial fibrillation and unmet needs of its management in Southeast Asia and South Asia. *Lancet Reg Health Southeast Asia*. 2025;34:100512. | Regional policy synthesis detailing the burden of atrial fibrillation, diagnostic gaps, and health system interventions across South and Southeast Asia. |
| XVIII | Adolescent Mental Health | Surenthirakumaran R, Kumaran S, Coonghe PAD. Prevalence of mental health problems and associated factors among the school students aged 13-15 years in Jaffna District, Sri Lanka. *J Coll Community Physicians Sri Lanka*. 2021;27(2):369–378. | Cross-sectional assessment using the Strengths and Difficulties Questionnaire (SDQ); identified a 21.5% prevalence of behavioral and emotional difficulties among adolescents. |
| XIX | Community Empowerment & Public Health | Shanmuganathan Y, Raguraman S, Kumaran S, Shiribavan K. Women empowerment is a tool to improve the quality of life of a community—case study. *Jaffna Med J*. 2023;35(1). | Evaluates participatory action research (PAR) frameworks utilizing women's empowerment as a structural mechanism to improve community quality of life in low-resource settings. |
| XX | Primary Care System Development | Subaschandren K, Surenthirakumaran R, Haq A, Nithiyalingam A, Gobith R. Public and patients involvement in chronic illness care, a way to provide universal health coverage in poor resource setting—a case study. *Jaffna Med J*. 2018;30(2). | Case study advocating for patient- and public-involved primary healthcare models to maintain continuous care for non-communicable diseases. |
| XI | Special Education & Caregiver Quality of Life | Sasrubi S, Lokubalasuriya L, Subaschandren K. The effect of socioeconomics, health and environmental factors on Quality of Life of Caregivers of students studying in special education in Jaffna District, Sri Lanka. *Sabaragamuwa Univ J*. 2025. | Quantitative study identifying socio-economic and institutional predictors of caregiver strain among families of neurodivergent children. |
| XII | Community Involvement & Autism | Shanmuganathan Y, Sasrubi S, Subaschandren K. The Implementing community co-designed model to enhance functioning in autistic children through skill development play activities. A case study Shanmuganathan. Asia Pac J Paediatr Child Health, Volume 7, Apr - Jun 2024 | The model showed viable to enhance the autism which can be applicable to similar settings. |
